# Discharge Planning and Home Care Needs Assessment for Older Patients in a Nursing Hospital

**DOI:** 10.3390/medicina56020060

**Published:** 2020-02-04

**Authors:** Lina Urbietė, Vita Lesauskaitė, Jūratė Macijauskienė

**Affiliations:** Geriatric Department, Medical Academy, Lithuanian University of Health Sciences, 44307 Kaunas, Lithuania; vita.lesauskaite@lsmuni.lt (V.L.); jurate.macijauskiene@lsmuni.lt (J.M.)

**Keywords:** nursing, home care needs, older people, health status, institutional nursing, InterRAI (HC)

## Abstract

*Background and objectives*: Following the accumulation of a sufficient amount of scientific evidence, it is now possible to appeal for changes in the organization of nursing services. Our aims are to assess the health status of patients discharged from nursing hospitals and to identify their home care needs by applying the international InterRAI Home Care (HC) assessment form. *Material and methods*: 152 geriatric patients (older than 65 years of age) discharged after a 90–120-day stay at a nursing hospital were examined using face-to-face interviews. The data from the medical records were also assessed. The capacities of patients were discussed with the patients themselves, nursing personnel, and relatives of the patients. *Results*: The analysis revealed that 45.4% of the respondents had severely impaired cognitive skills, while 27.6% had moderately impaired cognitive skills for decision making in daily living. People with greater cognitive difficulties were more dependent during daily instrumental activities and ordinary daily activities. The strongest relationship was established among the cognitive skills and management of medications, management of finances, and ordinary housework. For the greater part of respondents, a special need for permanent nursing (57.9%) or assistance (25.7%) was determined, i.e., official, state-funded nursing at home was appointed. The remaining respondents (16.4%) were not appointed further state-funded nursing or assistance at home, but an assessment of the independence of these patients based on the InterRai Activities of Daily Living Hierarchy Scale indicated that these skills varied from moderate independence (decision making was difficult only in new situations) to severely impaired skills (made no independent decisions or they were scarce). Despite the low independence of respondents, the majority of them would prefer nursing services at home to institutional nursing. *Conclusions*: The low independence observed in all participants, as well as their limited capacities, prove the need for nursing services at home and the necessity of their continuity. Despite the low independence of respondents, the majority of them would prefer nursing services at home to institutional nursing.

## 1. Introduction

Due to our aging society and multimorbidity, i.e., the coexistence of two or more chronic conditions in the same individual, which is common in an aging society [1,2], increasingly more geriatric patients are hospitalized and the length of their stay at hospitals is longer than that of younger patients. For the majority of older people, a longer stay at a hospital increases the reduction of their independence and decreases the possibility of restoring that independence [3,4]. Scientific research shows that discharge planning helps to shorten the length of the patients’ stay at treatment hospitals, assure the continuity of health care after discharge, avoid rehospitalization or premature acceptance into permanent care institutions, and increase the satisfaction of patients and their relatives with respect to health care services [5,6]. For these reasons alone, it is essential to make every effort to ensure that patients are treated in hospitals for the shortest time possible. It is necessary to assess their health status properly and to plan further nursing services at home.

In Lithuania, older people with the need for nursing services usually obtain these services at nursing hospitals that, unlike nursing homes in Germany, Denmark, the United Kingdom, and other European countries, are more oriented toward medical nursing and supportive treatment. In nursing hospitals, more attention is given to the performance of nursing procedures than to the planning of patients’ leisure time activities, communication with them, or the establishment of a home-like environment. Supportive treatment is the treatment of chronic diseases when the diagnosis is clear, active treatment is not required, and medical rehabilitation is contraindicated. If the patient contracts another disease (different than the disease for which the patient has been hospitalized in the nursing hospital) or if the chronic disease flares up, the patient is transferred to a clinical hospital. Based on their environment and provided services, nursing hospitals are more similar to clinical hospitals. The aim of nursing hospitals is to retain individual physical activity and to maintain the functional capacity of the organism, based on its potential. The nursing team is constituted of a physician (working in the nursing hospital), nurse, nurse’s assistant, physical therapist, and social worker [7]. It has been proven that this type of hospital not only stabilizes the condition of patients but also produces positive results for patients’ health status [8].

In Lithuania, the demand for nursing hospitals is constantly increasing; thus, the network of these hospitals is continuously extended and the number of nursing beds is increased. For instance, in 2001, 13.29 of this type of bed was provided per 10,000 residents, while in 2018, this number reached 20.48 beds [9]. In these hospitals, nursing services are covered by the State for 120 days per year [10]. This period can be viewed as a respite period for patient’s relatives after nursing him/her at home or as a preparation time for relatives that need to provide nursing services at home after acute conditions, i.e., when the patient suddenly becomes dependent, and after the hospitalization, requires nursing services. After this period ends, irrespective of their independence level and health status, patients are discharged home or, by the request of relatives, are transferred to private permanent care institutions where nursing expenses are paid for by the patients themselves or their family members. For patients requiring palliative care, the duration of these services in the hospital is not limited [11]. Persons for which, due to various social reasons, it is impossible to provide nursing and care services at home are accommodated at permanent care institutions. For these people, services at permanent care institutions are partially financed by the State. In contrast to nursing hospitals, the purpose of these institutions is to take care of older people.

The great demand for nursing services in hospitals is related to the system of nursing services at home that is still being developed and reorganized. Nursing services at home are provided only for those patients that have special needs for permanent nursing (SP) or special needs for permanent care (assistance) with considerable special needs. Considerable special needs are attested to by a valid certificate of special needs provided by the Disability and Working Capacity Assessment Office under the Ministry of Social Security and Labour of the Republic of Lithuania (DWCAO). These needs are established for an individual if the following conditions are confirmed: a general functional disorder is identified due to somatic illnesses or injuries; the score of the Barthel index is from 20 to 61 points and people are totally dependent or almost totally dependent on the assistance of other people in their daily activities; people that have had a tracheostomy or gastrostomy and whose mucus needs to be removed from the trachea [12]. If the need for permanent nursing or care is identified, the individual gains the right not only to certain nursing services at home and social assistance financed by the State but also to financial compensations. A person can use this compensation for nursing or care needs and for hiring a nurse or assistant.

While analyzing the data from DWCAO, it was noticed that the number of older people with an established need for permanent nursing is decreasing in Lithuania. From 2012 to 2016, this number decreased by approximately 1000 individuals each year (from 18,839 to 14,699). In 2017, a slightly greater reduction was visible in comparison to 2016 (2016—14,699, 2017—11,204). Despite these tendencies, the need for nursing services at home is not being met. Rosita Stasiukaitienė noticed that this reduction had appeared after a change in the procedure of the establishment of special needs [13]. Nevertheless, this does not mean that the number of people that need permanent nursing or assistance has decreased.

In Lithuania, the science and practice of nursing is a novel, dynamically developing field, grounding the directions and models of the practical nursing services organization. Following the accumulation of a sufficient amount of scientific evidence, it is possible to appeal for changes in the organization of nursing services that are validated by legislation from the Lithuanian government and orders from the Minister of Health. In Lithuania, there is scarce research that explores the health status of older people discharged from nursing hospitals, the nursing problems that they deal with, and the weak points of the nursing organization and suggestions for its improvement.

Our aims are to assess the health status of patients discharged from nursing units and to identify their home care needs by applying the international InterRAI (HC) assessment form.

## 2. Methods

A quantitative cross-sectional study design was implemented. The research was carried out from June 2017 to June 2018. Patients (older than 65 years of age) treated in a nursing and supportive treatment hospital for 90 to 120 calendar days and discharged home participated in the research. This period was selected based on the average length of stay at the hospital and the 120 calendar days that are covered by the State. This period can be used by the patients at once or in a phased manner. In our selected hospital, the average stay was 90 days. Limited research exists on the independence of discharged patients and their nursing problems. The population size of the research was identified based on five years of statistical data on patients that had been discharged from the nursing hospital.

The size of the representative sample, calculated with a 2% bias and 98% probability, was 152 patients. All patients that had been discharged after a 90–120-day hospital stay were invited to participate in the research. No one refused to participate. Thus, 152 patients were interviewed.

The method of the research was a face-to-face interview, conducted with patients in the supportive treatment and nursing hospital one week before their discharge. In addition, documented data on treatment and nursing, i.e., diagnoses, prescriptions by physicians (medications), and evaluations of the physical therapy specialist and social worker, were also assessed. The skills of patients were evaluated by discussing them with the patients, nursing personnel, and relatives of the patients.

The instrument of the research was the InterRAI Home Care (HC) Assessment System that was designed to be a user-friendly, reliable, person-centered system to inform and guide the comprehensive planning of care and services for elderly and disabled persons in community-based settings [14]. In order to translate the assessment form and adjust it to the Lithuanian language and culture, these main steps, described in scientific literature, were taken: translation (from English into Lithuanian); back-translation (from Lithuanian into English); comparison of texts in both languages; their coordination until they both fully coincided; and the assessment of the content validity. The assessment of the content validity determined if the assessment form was suitable for the reflection of features of the researched phenomena.

The cognition of respondents was assessed by employing questions from Section C (cognition) of the InterRAI HC assessment form related to cognitive skills, memory, thinking (easily distracted, episodes of disorganized speech, variation of mental function over the course of the day), change in mental status from a person’s usual functioning, and change in decision making as compared to 90–120 days ago. When assessing the independence of the patient based on cognitive skills, the status of the patient can vary from independent to severely impaired. Three memory types were assessed: short-term memory, procedural memory, and situational memory.

The functional status of respondents was analyzed by using questions from Section G (functional status) of the InterRAI HC assessment form. Instrumental activities of daily living (meal preparation, ordinary housework, managing medications, phone use, and walking up/down stairs) were assessed by questioning the nursing personnel that had taken constant care of the patient.

The performance of ordinary daily activities was assessed by observing the patient and by evaluating the nursing documentation. Bathing, personal hygiene, dressing the upper body, dressing the lower body, walking, toilet use, and eating were assessed.

The assessment of the daily activities questionnaire was carried out in accordance with ADLHS (Activities of Daily Living Hierarchy Scale) [15,16].

Four variables of daily activities were assessed:

1. Personal hygiene;
2. Toilet use;
3. Locomotion;
4. Eating.

Daily activities were assessed by points:

0. Independent.
1. Independent but with some setup help (e.g., laying out clothes).
2. Supervision but no direct hands-on support.
3. Limited assistance (help but not weight-bearing).
4. Extensive assistance (weight-bearing help but the person still performs 50% or more of subtasks).
5. Maximal assistance (weight-bearing support for more than 50% of subtasks).
6. Total dependence.

### 2.1. Statistical Methods

Cronbach’s alpha was calculated in order to assess the inner consistency of the assessment scale. In our case, this coefficient was 0.83 (Part C of the assessment form) and 0.95 (Part G of the assessment form).

Statistical data analysis was performed by employing the IBM SPSS Statistics^®^ Statistical Package for Social Sciences 20 (Armonk, NY, USA) for Windows and Microsoft Office Excel 2010 (Redmond, WA, USA).

The χ^2^ value or Fisher’s exact test was used to assess the interdependence of the qualitative features.

Spearman’s rank correlation coefficient was employed for analysis of the reciprocal correlations of categorical data.

The Friedman test was applied for the comparison of three or more dependent samples.

The selection of institutional nursing or nursing services at home was assessed using univariate logistic regression methods. We calculated the values of the relative risk (RR) and their 95% confidence intervals (CI).

While checking the statistical hypotheses, the selected significance level was 0.05.

### 2.2. Ethical Consideration

The research was carried out only after obtaining permission from the Kaunas Regional Biomedical Investigations Ethical Committee (No BE-2-36; 28-06-2017) and Governmental Data Security Inspection (No 2R-6655). Before interviewing the respondents (and/or their relatives), their consent to participate in the biomedical research was obtained.

## 3. Results

Among the 152 patients that participated in the research, the number of women (115, 75.7%) was greater than the number of men. The age of the respondents varied from 65 to 96 years, with an average age of 82.5 years (SD ± 7.9). The majority of the research subjects were 80–89-year-old respondents, and most of them were widowers (111, 73.0%). Before their stay in the supportive treatment and nursing hospital, 55 (36.2%) of the research subjects lived with their children (without their spouse), and 50 (32.9%) lived alone.

The general characteristics of the research subjects are presented in Table 1.

Multimorbidity was common in the respondents. Usually, patients suffered from cardiovascular (86.2%), neurological (84.9%), and psychiatric diseases (19.1%) (Table 2).

When assessing the overall dependency of the participants during their hospitalization in a nursing hospital and upon their discharge, for 64.4% (*n* = 98) of patients, the level of their independence remained the same, for 25.7% (*n* = 39) it improved, while for 9.9% (*n* = 15) it deteriorated.

While assessing the cognitive skills (Table 3) of patients upon their discharge from the nursing hospital, it was found out that 45.4% of patients had severely impaired cognitive skills, and 11.2% of patients were independent in their decision making. All patients had problems with short-term memory. Disordered thinking (easily distracted, episodes of disorganized speech, and variation in mental functions over the course of the day) was observed in 85.5% of patients. The mental status of the majority of the discharged patients, as well as their skills for decision making in daily living, did not change during the time spent in the hospital.

**Figure 1:**
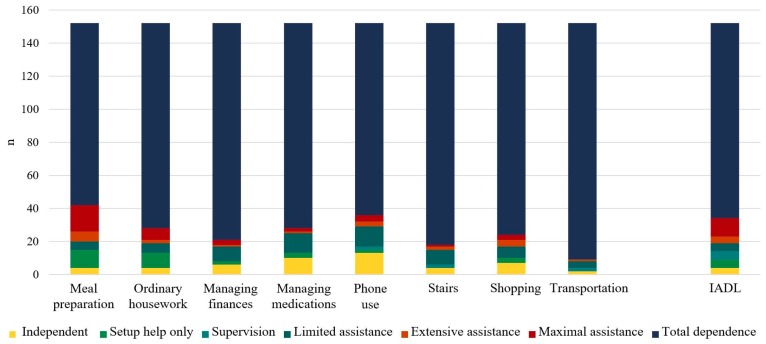
Capacity of the study subjects to perform daily activities. *n* = 152.

The skills to use public transport, walk up/down stairs, manage their finances, and shop were impaired the most (Figure 1).

The research revealed that people dealing with more serious cognitive difficulties were more dependent during their daily instrumental activities and ordinary daily activities (Spearman’s rank correlation for all activities was in range from 0.33 to 0.62, *p* < 0.0001). The strongest links were observed between the cognitive skills and medication management (*R* = 0.62), finance management (*R* = 0.54), and ordinary housework (*R* = 0.53). The weakest but still reliable link was revealed between cognitive skills and meal preparation (*R* = 0.33).

Upon discharging the patients from the nursing hospital, special needs (SN) were determined for our research subjects. Special needs for permanent nursing were identified for a major portion of the respondents (57.9%; *n* = 88), i.e., official, state-funded nursing services at home were appointed for them. Assistance services were appointed for 25.7% (*n* = 39) of patients. No official (state-funded) further nursing or assistance services at home were appointed for 16.4% (*n* = 25) of respondents.

Cognitive skills for decision making in daily living were moderately or severely impaired for the majority of our research subjects (they were dependent on assistance from others). Of the participants that did not receive nursing or assistance services at home, it was identified that only 5.9% were independent according to their cognitive skills for decision making in daily living. In other respondents, these skills varied from modified independence (decision making only difficult in new situations) to severely impaired skills (never or rarely made decisions independently) (Table 4).

After comparing the independence of the research subjects, determined according to the InterRai Activities of Daily Living Hierarchy Scale, with the established need for permanent nursing and assistance at home (intended continuity of official nursing, assistance services at home), it was revealed that for 3.3% of patients, further official state-funded nursing and assistance services at home were not planned, though the independence of the participants based on ADL was evaluated as 5 points. In other words, the respondents required the maximal assistance of two or more assistants, and the assistants performed 50% or more of all assignments (Table 5).

The determined independence of the research subjects based on the InterRai (HC) was compared with the established needs (planned continuity of services) in accordance with the procedures existing in Lithuania. The results revealed that 16.4% of participants had not been appointed official nursing/assistance services on the grounds of the procedures existing in Lithuania, but based on the InterRai HC assessment form, these services were of no use only to 3.9% of respondents (Table 6).

Despite the low independence of the respondents, the majority of them (*n* = 116; 76.3%) indicated that they would prefer nursing services at home instead of institutional nursing.

Only 23.7% (*n* = 36) of respondents would choose institutional nursing. By applying the univariate logistic regression, it was established that the selection of institutional nursing was related to the age, gender, and health status (diagnosis, cognitive level) of the respondents. The institutional nursing services were selected 2.7 times more often by respondents that belonged to the 65–84-year age group (CI 1.191–6.081; *p* = 0.017) and not by those that constituted the group 85 years old and older. In comparison with women, the selection of institutional nursing by men was statistically significant (OR 2.402; CI 1.083–5.327; *p* = 0.031).

The odds ratio that people with Alzheimer’s disease chose institutional care was 5.915 times greater than that for those that did not suffer from this disease (CI 1.342–26.071; *p* = 0.019).

The selection of institutional nursing also depended on the disorders of patients’ cognitive functions. Patients that had severely impaired cognitive skills for decision making chose institutional nursing more often (a statistically significant response) than those that had skills that were not impaired or were minimally impaired (Table 7).

## 4. Discussion

When comparing our research subjects as receivers of nursing services at home with the receivers of the same services from Canada, Germany, and other countries, in accordance with their age, gender, diseases, management of medications, and marital status, it was revealed that all of them were characterized by similar features. The age average was 82.5 years old, the majority of the subjects were single, and there were more women than men. Multimorbidity and polypharmacy were characteristic of the greater part of the respondents. Generally, patients suffered from cardiovascular, neurological, and psychiatric diseases [17,18,19]. Moreover, the majority of them had moderately or severely impaired cognitive skills for decision making in daily living. All patients had problems with short-term memory. The majority of them had disordered thinking (easily distracted, episodes of disorganized speech, and variation of mental function over the course of the day).

The health status and needs of the examined patients were similar to the results of other studies carried out by Lithuanian authors: the more the cognitive skills of respondents were impaired, the more they were dependent when performing their instrumental and ordinary daily activities; thus, the need for nursing services increased [20,21]. Similar research results were indicated by Tracy L. Mitzner et al. in their 2014 study [22].

When comparing the independence level of the research subjects, established by using the international InterRai HC assessment form, with the determined nursing and assistance needs, in accordance with the procedures existing in Lithuania, it was revealed that some patients had no official nursing and assistance services assigned for them at home and no planned support from family although the skills of the research subjects for decision making in daily living were severely or moderately impaired, and maximal assistance was required when performing ordinary daily activities. The obtained research results indicate that a limited length of stay in a nursing hospital does not solve nursing problems. The absolute majority of our patients had to receive continuous nursing or assistance services at home (to a lesser or greater degree) after being provided nursing services at a hospital. It is essential to identify the reasons why patients with a minimal level of independence are not provided with continuing official nursing/assistance services at home. In Lithuania, the established system for appointing nursing and assistance services at home needs to be reviewed and its shortcomings found and eliminated.

The usage of the international InterRai HC assessment form in our research exposed problems related to the planning of post-hospitalization nursing and assistance services. Other studies have revealed that the InterRai HC assessment form and the questionnaires of Instrumental Activities of Daily Living (IADL) and ordinary Activities of Daily Living (ADL) provide an appropriate and reasonable assessment of patients’ independence levels [16].

Despite the independence of the research subjects and existent nursing problems, the majority of the participants preferred to receive nursing services at home. The literature analysis reveals that this is common not only to the participants of our research but also to the majority of older people [23,24]. In Denmark, Germany, and Norway, the focus has shifted toward the development of nursing services at home and to the support of family members caring for their relatives. The goal is that older people should remain independent for as long as possible in their own environment, feeling comfortable and safe, while the nursing services at home are provided on time and in a qualitative manner; in this way avoiding hospitalization, both for clinical treatment or permanent nursing/care.

The main limitation of the study is that it reflects the data of one nursing hospital. The regulations of the discharge are the same in all nursing hospitals of Lithuania, but the capacities of patients and their preferences could slightly differ.

## 5. Conclusions

Low independence observed in all participants upon their discharge from the nursing hospital and their limited capacities prove the need for nursing services at home and the necessity of their continuity.

Despite the low independence of respondents, the majority of them would prefer nursing services at home to institutional nursing.

The InterRai HC assessment form is focused on a universal assessment of personal independence. Using this assessment, it is possible to indicate the independence problems that are encountered by people and the scope of required nursing and assistance services in a more precise manner.

The obtained research results reveal that the current setup procedures for appointing nursing/assistance services at home need to be reviewed and decisions made, ensuring the continuity of post-hospitalization nursing services at home.

## Figures and Tables

**Table 1:** Socio-demographic data of the study subjects.

Characteristics	*N* (Percent)
**Age groups (years):**	
65–74	23 (15.1)
75–84	60 (39.5)
≥85	69 (45.4)
**Gender:**	
Men	37 (24.3)
Women	115 (75.7)
**Marital status:**	
Not married	9 (5.9)
Married	28 (18.4)
Widowed	111 (73.0)
Divorced/separated	4 (2.7)
**Living arrangements before hospitalization:**	
Alone	50 (32.9)
With spouse/partner	23 (15.1)
With spouse/partner and other(s)	4 (2.6)
With children (without spouse/partner)	55 (36.2)
With parent or caregiver	1 (0.7)
With brother or sister	4 (2.6)
With another relative	10 (6.6)
Not with a relative	5 (3.3)

**Table 2:** Distribution of the study subjects based on the number of diseases and their group.

Number of Diseases:	*N* (Percent)
2–3	28 (18.5)
4–5	48 (31.6)
≥6	76 (49.9)
**Diseases**	
Cardiovascular diseases	131 (86.2)
Neurological diseases	129 (84.9)
Psychiatric diseases	29 (19.1)
Skeletal and connective tissue diseases	26 (17.1)
Musculoskeletal diseases	25 (16.4)
Diabetes	18 (11.8)
Diseases of eyes	12 (7.9)
Genitourinary diseases	12 (7.9)
Skin diseases	11 (7.2)
Digestive diseases	8 (5.3)
Oncological diseases	7 (4.6)
Other	19 (12.6)

**Table 3:** Cognitive skills of the study subjects discharged from the nursing hospital.

Cognitive Skills	*N* (Percent)
**Cognitive skills for daily decision making**	
Independent	17 (11.2)
Modified independence	15 (9.9)
Minimally impaired	9 (5.9)
Moderately impaired	42 (27.6)
Severely impaired	69 (45.4)
**Memory**	
Memory is good	19 (12.5)
Memory problems	133 (87.5)
Short-term memory problems	133 (100)
Procedural memory problems	120 (90.2)
Situational memory problems	123 (92.5)
**Thinking**	
Behavior not present	22 (14.5)
Behavior present, including:	130 (85.5)
Easily distracted	130 (100)
Episodes of disorganized speech	107 (82.3)
Mental function varies over the course of the day	104 (80.0)
**Acute changes in mental status from usual functioning of the person (e.g., restlessness, lethargy, difficulty waking up, altered environmental perception)**	
No change	132 (86.8)
Change	20 (13.2)
**Change in decision making over the last 90 days**	
Improved	14 (9.2)
No change	129 (94.1)
Declined	9 (5.9)

**Table 4:** Special needs (SN) and cognitive skills of the study subjects.

Established Special Needs (SN)	Todal									
	Independent		Modified/ Minimally Impaired		Moderately Impaired		Severely Impaired			
	*n*	%	*n*	%	*n*	%	*n*	%	*n*	%
No SN	9	5.9	9	5.9	5	3.3	2	1.3	25	16.4
Assistance	3	2.0	6	3.9	13	8.6	17	11.2	39	25.7
Permanent nursing	5	3.3	9	5.9	13	15.8	50	32.9	88	57.9
Total	17	11.2	24	15.7	42	27.6	69	45.4	152	100.0

χ^2^ = 36.0; *p* < 0.0001.

**Table 5:** Distribution of respondents based on the established special needs (SN) and independence level when performing ordinary activities of daily living (ADL).

Established Special Needs		Independence Score Based on the InterRai Activities of Daily Living Hierarchy Scale							Total
		0	1	2	3	4	5	6	
No SN	*n*	6	2	5	7	0	5	0	25
	%	3.9	1.3	3.3	4.6	0.0	3.3	0.0	16.4
Assistance	*n*	0	3	6	12	7	9	2	39
	%	0.0	2.0	3.9	7.9	4.6	5.9	1.3	25.7
Nursing	*n*	0	0	3	16	4	40	25	88
	%	0.0	0.0	2.0	10.5	2.6	26.3	16.4	57.9
Total	*n*	6	5	14	35	11	54	27	152
	%	3.9	3.3	9.2	23.0	7.2	35.5	17.8	100.0

χ^2^ = 67.8; *p* < 0.0001.

**Table 6:** Distribution of research subjects based on their independence level and continuity of intended services.

Service Continuity is Intended Upon Discharge	Nursing/Assistance	OfficialNnursing/Assistance Services Aare Not Intended	Significance
Established needs (according to the Lithuanian procedures)	127 (83.6)	25 (16.4%)	*p* < 0.001
Independence level according to the InterRAI HC assessment form	146 (90.1)	6 (3.9%)	

**Table 7:** Probability of choosing institutional nursing by patients having disorders of cognitive functions (univariate logistic regression).

Variable		OR (95%, CI)	*p*
Cognitive skills for Daily Decision Making	Severely impaired vs. independent, modified/minimally impaired *	2.680 (1.017–7.062)	0.046
Ability to be self-understood	More often unable to express themselves vs. more often able to express themselves *	2.199 (1.018–4.748)	0.045
Ability to understand others	More often do not understand others vs. more often understand others *	2.480 (1.137–5.410)	0.022
Behavior	Wandering vs. not wandering *	3.691 (1.163–11.716)	0.027
	Socially unacceptable behavior (verbal/physical abuse, inappropriate public sexual behavior or undressing, resistance to care)	3.262 (1.173–9.069)	0.023

* Reference group.
